# Association between maternal smoke exposure and congenital heart defects from a case–control study in China

**DOI:** 10.1038/s41598-022-18909-y

**Published:** 2022-09-02

**Authors:** Changfei Deng, Jie Pu, Ying Deng, Liang Xie, Li Yu, Lijun Liu, Xiujing Guo, Sven Sandin, Hanmin Liu, Li Dai

**Affiliations:** 1 Department for Women and Children’s Health, West China Second University Hospital, Sichuan University, Chengdu, 610041 Sichuan China; 2 Key Laboratory of Birth Defects and Related Diseases of Women and Children (Sichuan University), Ministry of Education, Chengdu, 610041 Sichuan China; 3 Department of Obstetrics and Gynecology, West China Second University Hospital, Sichuan University, Chengdu, 610041 Sichuan China; 4 National Center for Birth Defects Monitoring, West China Second University Hospital, Sichuan University, Chengdu, 610041 Sichuan China; 5 The Joint Laboratory for Pulmonary Development and Related Diseases, West China Institute of Women and Children’s Health, West China Second University Hospital, Sichuan University, Chengdu, 610041 Sichuan China; 6 NHC Key Laboratory of Chronobology, Sichuan University, Chengdu, 610041 Sichuan China; 7 Med-X Center for Informatics, Sichuan University, Chengdu, 610041 Sichuan China; 8 Department of Pediatric Cardiology, West China Second University Hospital, Sichuan University, Chengdu, 610041 Sichuan China; 9 Department of Medical Epidemiology and Biostatistics, Karolinska Institute, Stockholm, Sweden; 10 Department of Psychiatry, Icahn School of Medicine at Mount Sinai, New York, NY 10029 USA

**Keywords:** Risk factors, Environmental impact

## Abstract

There is a gap in knowledge how maternal exposure to environmental tobacco smoke (ETS) is associated with offspring congenital heart defects (CHDs). In this case–control study, we collected data on 749 fetuses with CHDs and 880 fetuses without any congenital anomalies to examine the association of maternal ETS with fetal CHDs and the potentially moderating effect by maternal hazardous and noxious substances (HNS), periconceptional folate intake and paternal smoking. Maternal exposure to ETS in first trimester was associated with increased risk of CHDs in a dose–response gradient, with the AORs (95% CI) were1.38 (1.00–1.92), 1.60 (1.07–2.41), and 4.94 (2.43–10.05) for ETS < 1 h/day, 1–2 h/day, and ≥ 2 h/day, respectively. With the doubly unexposed group as reference categories, AORs for maternal ETS exposure ≥ 2 h/day in the absence of folate intake, in the presence of HNS exposure or paternal smoking, were 7.21, 11.43, and 8.83, respectively. Significant additive interaction between ETS exposure and maternal folate intake on CHDs was detected. Maternal ETS exposure during first trimester may increase the risk of offspring CHDs in a dose–response shape, and such effect may be modified by maternal folate intake or other potential factors.

## Introduction

Worldwide, congenital heart defects (CHDs) are the most common birth defects and the major cause of death in fetuses and newborns, affecting about 4–50 cases per 1000 live births^1^. Previous studies indicated that fetus CHDs may result from nutritional and environmental factors, including maternal and paternal smoking^2^. World Health Organization (WHO) global report indicated 1.9% prevalence of smoking among women in China, aged 15 or higher, which is much lower than that in Europe (20.7%) and the USA (12.4%)^3^. However, more than 70% of Chinese women are exposed to passive smoking^4^. Some studies found an association between maternal smoking and offspring CHDs^5–7^, but only few provided evidences whether maternal exposure to environmental tobacco smoke (ETS) increases the risk of CHDs and the results keep inconsistent. Furthermore, the effects of exposure period, dose and interaction by maternal folate intake, maternal hazardous and noxious substances (HNS) exposure, paternal smoking remain to be explored.

In the present study, we hypothesize that offspring to mothers exposed to ETS are at increased risk of CHDs. Secondary hypotheses include the effect of timing of exposure and the effect of exposure dose. We also examine the interaction with parental factors.

## Results

### Demographic characteristics

The maternal, paternal, and infant characteristics of 749 CHD cases and 880 controls were presented in Table 1. Compared with control mothers, more case mothers aged less than 25 years of old, had 9 years of education or less, lived in rural area, and had a family history of congenital anomalies. There were much more case mothers who exposed to hazardous and noxious substances (HNS) from 3 months prior to conception through the first trimester than control mothers, but fewer took folic acid supplements. Furthermore, paternal smoking during 0–3rd months before conception were more frequently in case group than in control group.

**Table 1:** Subject characteristics of the CHD cases and controls.

Characteristics	Cases		Controls		*χ*^*2*^	*p* value
	No	%	No	%		
**Maternal age (years)**					114.03	< 0.001
< 25	167	22.30	46	5.23		
25–29	346	46.19	424	48.18		
30–34	170	22.70	304	34.55		
≥ 35	66	8.81	106	12.05		
**Maternal education level**					222.12	< 0.001
≤ 9 school years	142	18.96	27	3.07		
10–12 school years	153	20.43	51	5.80		
13–16 school years	392	52.34	649	73.75		
≥ 17 school years	62	8.28	153	17.39		
**Residence**					214.96	< 0.001
Urban	428	57.14	783	88.98		
Rural	321	42.86	97	11.02		
**Congenital anomalies family history**					13.66	< 0.001
No	645	86.11	808	91.82		
Yes	104	13.89	72	8.18		
**Maternal folate intake**^**a**^					52.83	< 0.001
Yes	309	41.26	522	59.32		
No	440	58.74	358	40.68		
**Maternal HNS exposureb**					38.99	< 0.001
No	445	59.41	651	73.98		
Yes	304	40.59	229	26.02		
**Paternal smoking**^**c**^					24.90	< 0.001
No	429	57.28	609	69.20		
Yes	320	42.72	271	30.80		

^a^Taking folic acid over 90 days or more during the 3 months before conception to the first trimester.

bMaternal HNS (maternal hazardous and noxious substances) exposure during the 3 months before conception to the first trimester.

^c^Paternal smoking during 0–3 months before conception.

### Association between maternal ETS exposure and CHDs

As shown in Table 2, maternal exposure to ETS from 12th month before pregnancy through first trimester was associated with an increased risk of CHD in offspring. The adjusted odds ratios (AOR (95% CI)) were 1.67 (1.28–2.18) for total CHD, 1.58 (1.18–2.12) for septal defects (SPD), 1.86 (1.21–2.84) for conotruncal defects (CTD), 1.69 (1.15–2.48) for left ventricle outflow tract obstruction (LVOTO), 1.82 (1.25–2.67) for right ventricle outflow tract obstruction (RVOTO) and 2.73 (1.65–4.51) for other CHDs groups.

**Table 2:** Association between maternal ETS and CHDs by CHD subgroup exposure duration.

Groups	Non-ETS (No.)	3rd–12th months before pregnancy		0–12th months before pregnancy		12th months before pregnancy to 1st trimester	
		No	AOR (95% CI)	No	AOR (95% CI)	No	AOR (95% CI)
Controls	504	73		127		176	
Total CHDs	356	37	0.86 (0.55, 1.35)	74	0.81 (0.57, 1.14)	282	1.67 (1.28, 2.18)
SPD	261	33	1.07 (0.66, 1.72)	53	0.81 (0.55, 1.20)	185	1.58 (1.18, 2.12)
CTD	93	15	1.57 (0.81, 3.03)	17	0.68 (0.37, 1.27)	71	1.86 (1.21, 2.84)
LVOTO	112	8	0.67 (0.30, 1.48)	23	0.80 (0.47, 1.37)	77	1.69 (1.15, 2.48)
RVOTO	109	13	0.98 (0.50, 1.93)	23	0.77 (0.45, 1.33)	95	1.82 (1.25, 2.67)
Other CHDs	52	4	0.94 (0.31, 2.83)	7	0.53 (0.22, 1.29)	60	2.73 (1.65, 4.51)

Adjusted by maternal age, maternal education level, residence, congenital anomalies family history, maternal folate intake, maternal HNS exposure, paternal smoking.

*ETS* environmental tobacco smoke, *AOR* adjusted odds-ratio, *CI* two-sided confidence interval, *SPD* septal defects, *CTD* conotruncal defects, *LVOTO* left ventricle outflow tract obstruction, *RVOTO* right ventricle outflow tract obstruction, *AVR* anomalous venous return.

There seemed to be a statistically significant dose–response relationship between maternal ETS and offspring CHD (Table 3). We did not observe the association for ETS < 1 h per day, but found significant associations for higher dose of ETS exposure (1–2 h per day, AOR = 1.60, 95% CI 1.07–2.41; ≥ 2 h per day, AOR = 4.94, 95% CI 2.43–10.05).

**Table 3:** Association between maternal ETS during first trimester and CHDs by CHD subgroup exposure dose.

Groups	Non-ETS (No.)	< 1 h per day		1–2 h per day		≥ 2 h per day	
		No	AOR (95% CI)	No	AOR (95% CI)	No	AOR (95% CI)
Controls	504	109		56		11	
Total CHDs	356	144	1.38 (1.00, 1.92)	81	1.60 (1.07, 2.41)	57	4.94 (2.43, 10.05)
SPD	261	93	1.30 (0.90, 1.88)	54	1.53 (0.97, 2.40)	38	4.87 (2.29, 10.33)
CTD	93	33	1.41 (0.83, 2.40)	21	1.72 (0.91, 3.25)	17	6.16 (2.44, 15.53)
LVOTO	112	39	1.41 (0.87, 2.27)	24	1.66 (0.91, 3.01)	14	4.63 (1.84, 11.64)
RVOTO	109	46	1.42 (0.88, 2.28)	29	1.73 (0.99, 3.03)	20	6.07 (2.54, 14.53)
Other CHDs	52	31	2.12 (1.14, 3.94)	18	3.06 (1.51, 6.20)	11	10.52 (3.65, 30.29)

Adjusted by maternal age, maternal education level, residence, congenital anomalies family history, maternal folate intake, maternal HNS exposure, paternal smoking.

Similar dose-dependent gradients were also found in the associations between ETS exposure and CHD subtypes. Nearly all the strong associations were found for exposure to ETS ≥ 2 h per day within each CHD subtype, such as the AOR (95% CI) of 4.87 (2.29–10.33) for SPD: 6.16 (2.44–15.53) for CTD, 4.63 (1.84–11.64) for LVOTO, 6.07 (2.54–14.53) for RVOTO, and 10.52 (3.65–30.29) for other CHDs. (Table 3).

### Interaction between maternal ETS and parental factors

Table 4 presents the interactions of maternal ETS exposure (as four-level categorical variable) with maternal HNS exposure, and with paternal smoking (as binary variables). The highest AORs were observed for maternal ETS exposure ≥ 2 h per day in the absence of folate intake (AOR = 7.21, 95% CI 2.64–19.67), in the presence of HNS (AOR = 11.43, 95% CI 3.32–39.44), and in the presence of paternal smoking (AOR = 8.83, 95% CI 2.99–26.02) when taking the doubly unexposed group as reference categories in each subgroup analysis. There was significant additive interaction between maternal ETS exposure < 1 h/day and folate intake on CHDs after adjusting for potential confounders, with the relative risk due to the interaction (RERI) of 1.14 (95% CI 0.05–2.23), the attributable proportion due to interaction (AP) of 0.47 (95% CI 0.17–0.78), and the synergy index (SI) of 5.28 (95% CI 0.47–58.99). We also identified a significant multiplicative interaction on risk of CHD between maternal folate intake and exposure to ETS < 1 h/day (1.90, 95% CI 1.01–3.58). The additive interaction was further confirmed by other models with adjustment for various covariates. (Supplementary Tables S1–S3) However, no significant additive interaction on CHDs was found between maternal ETS and other two parental factors. When taking each pair of exposures as binary variables in the analyses, two of indicators measuring the additive interaction between maternal ETS exposure and folate intake remained still significant (RERI = 1.03, 95% CI 0.10–1.95; AP = 0.39, 95% CI 0.12–0.66; SI = 2.72, 95% CI 0.90–8.18) (Supplementary Table S4), even after adjusting for different covariates (Supplementary Table S5). In subgroup analyses, a positive interaction between maternal ETS exposure and folate intake was found in group without congenital anomalies family history (RERI = 1.62, 95% CI 0.38–2.87; AP = 0.44, 95% CI 0.21–0.67; SI = 2.53, 95% CI 1.23–5.22), or in urban group (RERI = 1.01, 95% CI 0.04–1.97; AP = 0.41, 95% CI 0.11–0.71; SI = 3.32, 95% CI 0.70–15.64) after adjusting for maternal HNS exposure and paternal smoking. There seemed to be a positive interaction between maternal ETS and HNS exposures in urban group (RERI = 1.09, 95% CI 0.02–2.16; AP = 0.42, 95% CI 0.11–0.73; SI = 3.22, 95% CI 0.76–13.67), and in the group of maternal education years > 12 years (RERI = 1.03, 95% CI 0.001–2.05; AP = 0.41, 95% CI 0.10–0.72; SI = 3.18, 95% CI 0.74–13.58).

**Table 4:** Interaction between maternal ETS exposure and parental factors on the risk of CHDs.

Groups	ETS dose				ORs (95% CI) for ETS within strata of another exposure		
	None	< 1 h/day	1–2 h/day	≥ 2 h/day	< 1 h/day	1–2 h/day	≥ 2 h/day
**Folate intake**^**a**^							
Yes	Ref	1.01 (0.64, 1.59)	1.56 (0.86, 2.83)	4.28 (1.57, 11.67)^#^	1.01 (0.64, 1.59)	1.56 (0.86, 2.83)	4.28 (1.57, 11.67)^#^
No	1.26 (0.93, 1.70)	2.41 (1.51, 3.85)^$^	2.09 (1.20, 3.64)^#^	7.21 (2.64, 19.66)^$^	1.92 (1.20, 3.06)^#^	1.67 (0.96, 2.89)	5.74 (2.11, 15.63)^$^
ORs (95% CI) for folate intake within strata of ETS	1.26 (0.93, 1.70)	2.38 (1.36, 4.16)^#^	1.34 (0.63, 2.85)	1.68 (0.42, 6.68)			
RERI (95% CI)		1.14 (0.05, 2.23)*	0.28 (− 1.13, 1.68)	2.67 (− 5.52,10.86)			
AP (95% CI)		**0.47 (0.17, 0.78)**^**#**^	0.13 (− 0.49, 0.76)	0.37 (− 0.47,1.12)			
SI (95% CI)		5.28 (0.47, 58.99)	1.34 (0.29, 6.09)	1.76 (0.34, 9.03)			
Multiplicative scale (95% CI)		1.90 (1.01, 3.58)*	1.07 (0.48, 2.40)	1.34 (0.33, 5.49)			
**HNS exposure**^**b**^							
No	Ref	1.09 (0.71, 1.68)	1.72 (1.05, 2.83)*	3.49 (1.45, 8.42)^#^	1.09 (0.71, 1.68)	1.72 (1.05, 2.83)*	3.49 (1.45, 8.42)^#^
Yes	1.27 (0.90, 1.79)	2.43 (1.54, 3.84)^$^	1.83 (0.95, 3.54)	11.43 (3.32, 39.44)^$^	1.91 (1.15, 3.18)^#^	1.44 (0.72, 2.89)	8.99 (2.55, 31.71)^#^
ORs (95% CI) for HNS within strata of ETS	1.27 (0.90, 1.79)	2.23 (1.27, 3.89)^#^	1.06 (0.48, 2.33)	3.27 (0.73, 14.57)			
RERI (95% CI)		1.07 (− 0.07, 2.21)	− 0.16 (− 1.63, 1.30)	7.67 (− 6.70, 22.03)			
AP (95% CI)		0.44 (0.11, 0.77)^#^	− 0.09 (− 0.94, 0.76)	0.67 (0.19, 1.15)^#^			
SI (95% CI)		3.93 (0.60, 25.71)	0.83 (0.16, 42.38)	3.77 (0.67, 21.30)			
Multiplicative scale (95% CI)		1.75 (0.91, 3.36)	0.84 (0.36, 1.96)	2.57 (0.56, 11.90)			
**Paternal smoking**^**c**^							
No	Ref	1.48 (0.95, 2.32)	1.41 (0.84, 2.37)	3.04 (1.16, 7.99)*	1.48 (0.95, 2.32)	1.41 (0.84, 2.37)	3.04 (1.16, 7.99)*
Yes	1.07 (0.75, 1.53)	1.44 (0.96, 2.18)	2.10 (1.13, 3.89)*	8.83 (2.99, 26.02)^$^	1.35 (0.83, 2.20)	1.97 (1.01, 3.83)*	8.26 (2.72, 25.11)^$^
ORs(95% CI)	1.07 (0.75, 1.53)	0.97 (0.56, 1.69)	1.49 (0.69, 3.22)	2.90 (0.70,12.10)			
RERI (95% CI)		− 0.10 (− 1.03, 0.84)	0.66 (− 0.82, 2.15)	6.10 (− 4.49, 16.69)			
AP (95% CI)		− 0.06 (− 0.69, 0.56)	0.31 (− 0.24, 0.86)	0.65 (0.14, 1.15)^#^			
SI (95% CI)		0.84 (0.18, 4.02)	2.35 (0.32,17.35)*	3.59 (0.60, 21.55)^$^			
Multiplicative scale (95% CI)		0.92 (0.48, 1.77)	1.42 (0.61, 3.31)	2.72 (0.63, 11.82)			

*p < 0.05; ^#^p < 0.01; ^$^p < 0.001.

^a^Adjusted by maternal age, maternal education level, residence, congenital anomalies family history, maternal HNS exposure, paternal smoking.

^b^Adjusted by maternal age, maternal education level, residence, maternal folate intake, congenital anomalies family history, paternal smoking.

^c^Adjusted by maternal age, maternal education level, residence, maternal folate intake, congenital anomalies family history, maternal HNS exposure.

We further analyzed the interactions on the risks of five CHD subtypes by including each pair of exposures as dichotomous variables into the models, and found inconsistent results. As shown in Supplementary Tables S6–S8, statistically significant positive interactions between maternal ETS exposure and not taking folate intake were found on the risk of SPD (RERI = 1.26, 95% CI 0.07–2.45; AP = 0.37, 95% CI 0.10–0.63; SI = 2.06, 95% CI 1.02–4.16), RVOTO (RERI = 1.99, 95% CI 0.17–3.81; AP = 0.45, 95% CI 0.17–0.72; SI = 2.36, 95% CI 1.06–5.26), and other CHDs (RERI = 3.31, 95% CI 0.69–5.92; AP = 0.61, 95% CI 0.35–0.86; SI = 3.92, 95% CI 1.16–13.17) respectively, with the adjustment for maternal HNS exposure and paternal smoking. A possible positive additive interaction was found between maternal ETS exposure and paternal smoking on the risk of RVOTO (RERI = 1.40, 95% CI 0.09–2.72; AP = 0.46, 95% CI 0.13–0.79; SI = 3.16, 95% CI 0.73–13.70) after adjusting for maternal folate intake and HNS exposure. When other covariates (maternal age, education level, residence, and congenital anomalies family history) were included in the models, the estimates for some indicators became no longer statistically significant, and some invalid estimates were generated.

## Discussion

In this study, we observed that maternal ETS exposure in first trimester may increase the risk of CHD in the offspring, in a dose–response shape. In addition, the risks of CHDs or some subtypes were modified by intake of maternal folate supplement, HNS exposure and paternal smoking.

Our findings are consistent with previous studies^8,9^. Tobacco smoke contains a large amount of toxins and carcinogens, including nicotine, carbon dioxide, carbon monoxide, carbonyls, etc. Nicotine and carbon monoxide both rapidly crosses the placenta, with chronic exposure, may contribute to fetal hypoxia^10^. Pregnant rats exposed to a mild concentration of carbon monoxide may delay postnatal electrophysiological maturation of ventricular myocytes from newborns rats, likely vulnerable to life-threatening arrhythmias^11^. Moreover, passive smoking contains elevated concentrations of harmful materials, perhaps even higher than for active smoking^12,13^. Both spermatozoa and the oocytes can be vulnerable to DNA damage due to tobacco smoke exposure^14,15^. Accumulating evidence demonstrate that maternal smoking before and after pregnancy as well as passive smoking may result in miscarriage, stillbirth, low birth weight, childhood asthma development and offspring CHDs^16–19^.

In our study, maternal exposure to ETS from 12th months before pregnancy through first trimester was associated with increased risk of CHDs in offspring, while such association was not for maternal ETS exposure during 0–12th months before pregnancy. Likewise, in a National Birth Defects Prevention Study, modest positive association were detected between maternal exposure to secondhand tobacco smoke during the period one month before conception through the first trimester and some CHD subtypes (conotruncal defects, aortic stenosis and atrial septal defects)^19^. In first trimester, the key period of fetal heart development and teratogenesis, fetal developing heart is vulnerable to hazardous and noxious stimuli resulting in cardiovascular anomalies^20^. Most CHDs occur in fetuses between 2 and 9 weeks of gestation, but many women are not aware of being pregnant during this period^21^. Fetal and neonatal health outcomes improves if mothers keeping away from the tobacco smoking or exposure early in pregnancy.

Our study further provided evidence of the dose–response relationships between maternal ETS exposure during first trimester and CHDs. The dose-dependent associations of maternal active and passive smoking on the risk of CHDs were identified in previous studies^6,22,23^. By testing the hair nicotine concentration (HNC), Li et al. found^9^ that compared with the low concentration group, the associations became sharply stronger in higher HNC on the risk of CHDs.

It has been generally recognized that periconceptional maternal folate intake may reduce the risk of CHDs^24,25^. In China, rural women can be offered folic acid freely if they plan to be pregnant since ‘Folic acid for the prevention of neural tube defects’ was launched in 2009. Although the proportion of women consuming folate intake supplements is increasing, many women did not take folate supplements during three months before and after conception. Over 50% of male adults smoke in China^26^, which contributes to high exposure of passive smoking, also for pregnant women. Moreover, due to the placental barrier is not completely impermeable to the passage of environmental harmful chemical contamination^27^, maternal HNS exposure during the first trimester represents the most critical window of exposure for CHDs^28–30^. In this study, both maternal not taking folic supplements and HNS exposure were identified as risk factors of CHDs. We obtained strong evidence of additive interactions between maternal folate intake and ETS exposure on CHDs and several subtypes, while the evidence of interaction between maternal HNS, paternal smoking and ETS exposures were relatively weak as such effects were only observed for some CHD subtypes in stratification analyses. The findings highlight the significance of maternal folic acid supplementation and staying away from toxic and harmful environment to reduce the risk of CHDs as well as other adverse outcomes in offspring.

Our study has several strengths. First, all CHDs, including stillbirth, aborted pregnancy and livebirths, were collected to minimize the potential selection bias. Second, all CHD cases were diagnosed by echocardiography test and excluded syndromic or chromosome abnormality. Livebirths with CHDs were followed up by one year after deliver to validate the diagnosis results. The classification of CHD subtypes was used into relatively homogeneous subgroups to present the underlying etiologies and the potential risk factors under study to maximize the potential for meaningful analysis. Detailed information about maternal ETS exposure and paternal and maternal smoking makes it possible to comprehensively estimate the effect of exposure on CHD. Third, the epidemiological characteristics were collected in first trimester of pregnancy so recall bias could be very minimal.

The study also has limitations. The study was conducted in one hospital only, so replication is needed. Still, West China Second University Hospital is a large and high quality hospital and each year over 15,000 deliveries are recorded. Maternal ETS and HNS exposure were based on self-report, without testing biomarker levels. Further researches are warranted to better understand the molecular basis of CHD as well as the influence of gene-environment interactions.

## Conclusion

Maternal ETS exposure during first trimester may increase the risk of CHD in the offspring in a dose–response shape, and the risk maybe modified by maternal folate intake, HNS exposure, and paternal smoking. These findings highlight the importance of prescheduled pregnancy and prevention, including folic acid supplementation, paternal smoking cessation, maternal avoidance from ETS and HNS at home or workplace, etc. Preventive strategies to reduce the occurrence of major CHDs are necessary for those pregnant women or families exposed to ETS in the presence of other risk factors.

## Materials and methods

### Study population and data collection

This case–control study was performed at the West China Second University Hospital from January 2014 to December 2016. The hospital serves the entire south western part of China, and Tibet, and ranks in the top ten in both obstetrics and gynecology and pediatric in China^31^. Study inclusion criteria were singleton pregnancy, and for cases non-syndromic CHDs (Code Q20-26 by ICD-10), which referred to cases with CHDs only and without other non-cardiac anomalies, and for controls, no CHDs or other congenital malformations. Cases and controls with extra-cardiac abnormalities, syndromic diseases and chromosomal aberrations, or whose mothers were active smokers were excluded.

After providing signed informed consent, all participants were face-to-face interviewed in first, second, third trimester and one month after delivery^32^. The information of questionnaires included parental socioeconomic characteristics, congenital anomalies family history, maternal folic acid supplementation, exposure to ETS or HNS at home or workplace, etc.

The study was approved by the Ethics Committee of West China Second University Hospital and was based on the tenets of the Declaration of Helsinki.

### Case classification

CHDs were diagnosed via prenatal systematically echocardiography. Livebirths were confirmed via routine examination, which included heart auscultation and a neonatal echocardiography within one week after birth. Stillbirths diagnosed with CHD were aborted according to standard process. Each CHD medical record was reviewed by specialists, neonatal cardiologists to confirm diagnostic results. All CHD cases were encoding according to International Classification of Diseases-10 (ICD-10).

CHD cases were divided into five subtypes based on the anatomic lesion as follows: (1) septal defects (SPD), including atrial septal defects, ventricular septal defects, and endocardial cushion defects; (2) conotruncal defects (CTD), including transposition of the great arteries, tetralogy of Fallot, truncus arteriosus, and double outlet right ventricle; (3) left ventricle outflow tract obstruction (LVOTO), including aortic valve stenosis, hypoplastic left heart syndrome and variants, coarctation of the aorta, and interrupted aortic arch; (4) right ventricle outflow tract obstruction (RVOTO), including pulmonary valve stenosis, pulmonary atresia, tricuspid atresia, and Ebstein anomalies; (5) other CHDs, including anomalous venous return (total and partial anomalous pulmonary or systematic venous return), heterotaxia, and other cardiac structural abnormalities.

### Exposure measurements

**Figure 1:**
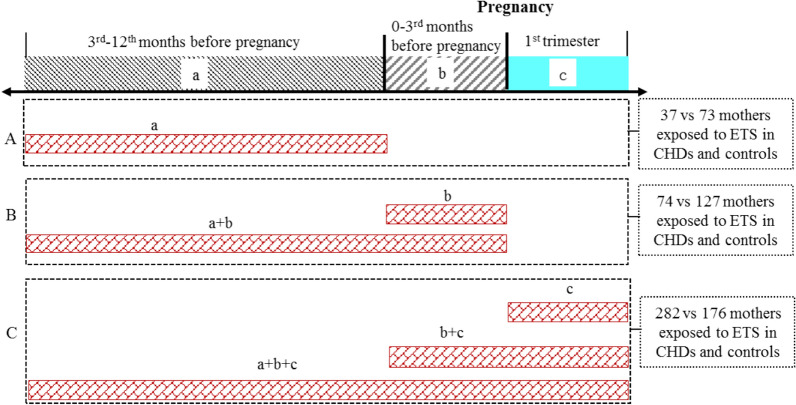
Exposure duration definition.

Maternal Environmental Tobacco Smoke (ETS) was measured through maternal self-report and was defined as exposure to tobacco smoke, at least an average of 15 min/day at home or workplace during 12rd before pregnancy to the first trimester^33^. There were three different exposure periods in: (a) 3rd–12th months before pregnancy, (b) 0–3rd months before pregnancy, and (c) the first trimester. We further created a categorical variable capturing duration of ETS exposure as (A) Equals interval (a) above; (B) Cumulative exposure from intervals (a) and (b); and (C) Cumulative exposure from intervals (a), (b) and (c) (Fig. 1). Maternal ETS exposure average dose during first trimester of pregnancy was classified into three groups: (1) less than 1 h per day; (2) 1–2 h per day; 3) 2 h or more per day.

Maternal exposure to HNS during 3rd months before conception through the first trimester was measured through self-report, including lead, cadmium, mercury, oil paint, solder, pesticide, insecticide, formaldehyde or other chemical materials.

Maternal folate intake means mothers taking folic acid supplement over 90 days or more during the 3rd months before conception to the first trimester^32,34^.

### Statistical analysis

We estimated the association between maternal ETS and fetal CHDs, including dose–response relationship, by calculating odds-ratios (OR) and two-sided 95% Wald-type confidence intervals from unconditional logistic regression^35^. First, we fitted a model only including a categorical covariate for maternal ETS and the nested categorical covariates for exposure interval and exposure dose. Second, we examined confounding by additionally adjusting for potential confounders, such as maternal age (< 25, 25–29, 30–34, and ≥ 35 years), maternal education level (≤ 9, 10–12, 13–16, and ≥ 17 school years), residence (urban vs rural), congenital anomalies family history (yes vs no), maternal folate intake (yes vs no), maternal HNS exposure (yes vs no), paternal smoking (yes vs no), if differed significantly between cases and controls examined by using Pearson Chi-square test. Third, we included parameters for interaction between maternal ETS exposure and each of covariates (maternal folate intake, maternal HNS exposure and paternal smoking), and assessed the individual and joint effects of the risk factors. Fourth, we evaluated the interactions between ETS and another risk factor (maternal folate intake, HNS exposure, and paternal smoking) on multiplicative scale, and additive scale as proposed by Knol and Vander Weele^36^. The relative excess risk due to interaction (RERI), attributable proportion due to interaction (AP), and synergy index (SI) with corresponding 95% CIs, were derived from the regression coefficients and covariance matrix from the multivariate logistic regression analyses by using an R function for additive interaction measures^37^, or the epiR and InteractionR package^38^. The 95% CI of the RERI or AP equal 0 and that of the SI equal 1 were defined as no additive interaction.

Last, we performed several sensitivity and subgroup analyses to assess the robustness of the interactive effects. (a) We compared the results of models which included ETS exposure as a multi-categorical variable with those of models including ETS as a binary variable. (b) To assess the effects of potential confounders, we adjusted for various covariates in the models (i.e., without any adjustment, adjusted for the two of three exposures when assessing the interactive effect of another one, fully adjusted by all the aforementioned covariates in models). (c) Subgroup analyses were performed across different groups stratified by maternal age (< 30 years vs ≥ 30 years), maternal education level (≤ 12 years vs > 12 years), residence (urban vs rural) and congenital anomalies family history (Yes vs No).

The statistical significance level for α was set at 0.05. And all analyses were performed using the R program 4.1.2.

## Supplementary Information

Supplementary Tables.
