# Supplementary material for: Association between maternal smoke exposure and congenital heart defects from a case–control study in China
Source: Sci Rep. 2022 Sep 2;12:14973. doi: 10.1038/s41598-022-18909-y (PMC9440088; doi:10.1038/s41598-022-18909-y)
Supplement: Supplementary file 1 — Supplementary Tables. [file 41598_2022_18909_MOESM1_ESM.pdf]

Supplementary Table S1 Interaction between maternal ETS exposure and parental factors on the risk of CHDs without adjustment for covariates

Groups	ETS dose				ORs (95%CI) for ETS within strata of another exposure		
	None	< 1 h/day	1-2 h/day	≥2 h/day	< 1 h/day	1-2 h/day	≥2 h/day
Folate intake							
Yes	Ref.	1.42(0.94,2.15)	1.70(0.97,2.99)	6.32(2.49,16.07) [$]	1.42(0.94,2.15)	1.70(0.97,2.99)	6.32(2.49,16.07) [$]
No	1.82(1.39,2.40) [$]	4.30(2.84,6.52) [$]	3.87(2.34,6.37) [$]	14.04(5.41,36.44) [$]	2.36(1.56,3.57) [$]	2.12(1.29,3.49) [#]	7.70(2.97,19.98) [$]
ORs (95% CI) for folate intake within strata of ETS	1.82(1.39,2.40) [$]	3.02(1.80,5.07) [$]	2.27(1.13,4.58) [*]	2.22(0.60,8.19)			
RERI (95%CI):		**2.06(0.34,3.77)** [#]	1.34(-0.69,3.37)	6.89(-7.48,21.27)			
AP (95%CI):		**0.48(0.23,0.72)** [$]	0.35(-0.05,0.74) [*]	0.49(-0.14,1.12)			
SI (95%CI):		**2.65(1.27,5.54)** [#]	1.88(0.76,4.65)	2.12(0.53,8.41)			
Multiplicative scale (95%CI):		1.66(0.92,2.98)	1.25(0.59,2.65)	1.22(0.32,4.62)			
HNS exposure							
No	Ref.	1.43(0.98,2.09)	1.93(1.22,3.05) [#]	5.18(2.31,11.63) [$]	1.43(0.98,2.09)	1.93(1.22,3.05) [#]	5.18(2.31,11.63)
Yes	1.55(1.14,2.11) [#]	3.27(2.17,4.93) [$]	3.10(1.72,5.60) [$]	16.47(4.98,54.45) [$]	2.10(1.33,3.33) [$]	2.00(1.07,3.73) [*]	10.61(3.15,35.69) [$]
ORs (95% CI) for HNS within strata of ETS	1.55(1.14,2.11) [#]	2.28(1.37,3.80) [$]	1.61(0.79,3.28)	3.18(0.76,13.22)			
RERI (95%CI):		1.28(-0.11,2.67)	0.62(-1.37,2.61)	10.74(-9.27,30.75)			
AP (95%CI):		0.39(0.09,0.70) [#]	0.20(-0.35,0.75)	0.65(0.17,1.13) [#]			
SI (95%CI):		2.30(0.95,5.56)	1.42(0.49,4.06)	3.27(0.70,15.16)			
Multiplicative scale (95%CI):		1.47(0.81,2.66)	1.03(0.48,2.25)	2.05(0.48,8.80)			
Paternal smoking							
No	Ref.	1.79(1.19,2.69) [#]	1.81(1.13,2.9) [*]	3.54(1.44,8.73) [#]	1.79(1.19,2.69) [#]	1.81(1.13,2.90) [*]	3.54(1.44,8.73) [#]
Yes	1.47(1.07,2.03) [*]	2.27(1.57,3.28) [$]	3.02(1.72,5.3) [$]	15.89(5.63,44.87) [$]	1.54(1.00,2.38) [*]	2.05(1.12,3.76) [*]	10.78(3.72,31.24) [$]
ORs (95%CI)	1.47(1.07,2.03) [*]	1.27(0.77,2.09)	1.67(0.83,3.36)	4.48(1.15,17.43) [*]			
RERI (95%CI):		0.01(-1.10,1.11)	0.74(-1.12,2.60)	11.87(-4.83,28.57)			
AP (95%CI):		0.00(-0.48,0.49)	0.25(-0.26,0.75)	0.75(0.42,1.07) [$]			
SI (95%CI):		1.00(0.42,2.40)	1.58(0.53,4.66)	4.94(1.08,22.61) [*]			
Multiplicative scale (95%CI):		0.86(0.47,1.56)	1.13(0.53,2.45)	3.04(0.75,12.28)			

Note: * p<0.05; # p<0.01; $ p<0.001.

Supplementary Table S2 Interaction between maternal ETS exposure and parental factors on the risk of CHDs with adjustment for selected covariates

Groups	ETS dose				ORs (95%CI) for ETS within strata of another exposure		
	**None**	**< 1 h/day**	**1-2 h/day**	**≥2 h/day**	**< 1 h/day**	**1-2 h/day**	**≥2 h/day**
Folate intake [a]							
Yes	Ref.	1.17 (0.76, 1.79)	1.64 (0.93, 2.90)	5.08 (2.01, 13.32) $	1.17 (0.76, 1.79)	1.64 (0.93, 2.90)	5.08 (2.01, 13.32) $
No	1.77 (1.34, 2.34) $	3.34 (2.16, 5.16) $	3.19 (1.91, 5.32) $	10.75 (4.09, 28.27) $	1.89 (1.23, 2.90) #	1.80 (1.08, 2.99) *	6.07 (2.31, 15.91) $
ORs (95% CI) for folate intake within strata of ETS	1.77 (1.34, 2.34) $	2.86 (1.69, 4.83) $	1.34 (0.63, 2.85)	1.94 (0.95, 3.96)			
RERI (95%CI):		**1.40 (0.02, 2.78)** *	0.78 (-0.99, 2.55)	4.80 (-5.42,16.01)			
AP (95%CI):		**0.42 (0.14, 0.70)** #	0.24 (-0.22, 0.71)	0.45(-0.23,1.13)			
SI (95%CI):		**2.49 (1.06, 5.88)** *	1.55 (0.59, 4.10)	1.97 (0.48, 8.12)			
Multiplicative scale (95%CI):		1.62 (0.89, 2.92)	1.10 (0.51, 2.35)	1.17 (0.31, 4.49)			
HNS exposure [b]							
No	Ref.	1.28 (0.86, 1.90)	1.81 (1.13, 2.90) *	4.43 (1.94, 10.11) #	1.28 (0.86, 1.90)	1.81 (1.13, 2.90) *	4.43 (1.94, 10.11) #
Yes	1.58 (1.15, 2.15) #	2.88 (1.88, 4.40) $	2.50 (1.37, 4.57) #	13.70 (4.10, 45.78) $	1.83(1.14, 2.94) #	1.59 (0.84, 3.51)	8.69 (2.55, 29.60) $
ORs (95% CI) for HNS within strata of ETS	1.58 (1.15, 2.15) #	2.25 (1.34, 3.79) #	1.38 (0.67, 2.85)	3.09 (0.73, 13.02)			
RERI (95%CI):		1.03 (-0.24, 2.30)	0.11 (-1.60, 1.82)	8.69 (-8.10, 25.48)			
AP (95%CI):		0.36 (0.03, 0.69) *	0.05 (-0.62, 0.71)	0.63 (0.13, 1.14) #			
SI (95%CI):		2.20 (0.83, 5.82)	1.08 (0.34, 5.48)	3.17 (0.66, 15.21)			
Multiplicative scale (95%CI):		1.43 (0.78, 2.62)	0.88 (0.40, 1.93)	1.96 (0.45, 8.55)			
Paternal smoking [c]							
No	Ref.	1.61 (1.06, 2.45) *	1.65 (1.02, 2.68) *	3.15 (1.26, 7.92) *	1.61 (1.06, 2.45) *	1.65 (1.02, 2.68) *	3.15 (1.26, 7.92) *
Yes	1.35 (0.97, 1.87)	1.90 (1.30, 2.77) $	2.47 (1.39, 4.39) #	13.10 (4.60, 37.33) $	1.41 (0.90, 2.20)	1.83 (0.98, 3.41)	9.71 (3.32, 28.71) $
ORs (95%CI)	1.35 (0.97, 1.87)	1.18 (0.70, 1.97)	1.50 (0.73, 3.06)	4.16 (1.05, 16.44) *			
RERI (95%CI):		-0.04 (-1.11, 1.03)	0.54 (-1.13, 2.20)	10.57 (-4.65, 25.78)			
AP (95%CI):		-0.02 (-0.53, 0.50)	0.21 (-0.10, 0.52)	0.74 (0.39, 1.09) #			
SI (95%CI):		0.97 (0.37, 2.51)	1.51 (0.44, 5.13)	4.78 (0.98, 23.27)			
Multiplicative scale (95%CI):		0.87 (0.48, 1.60)	1.13 (0.52, 2.46)	3.12 (0.76, 12.73)			

Note: * p<0.05; # p<0.01; $ p<0.001. a Adjusted by maternal HNS exposure and paternal smoking. b Adjusted by maternal folate intake and paternal smoking. c Adjusted by maternal folate intake and HNS exposure.

Supplementary Table S3 Interaction between maternal ETS exposure and parental factors on the risk of CHDs with adjustment for maternal age, residence, education level, and congenital anomalies family history

Groups	ETS dose				ORs (95%CI) for ETS within strata of another exposure		
	**None**	**< 1 h/day**	**1-2 h/day**	**≥2 h/day**	**< 1 h/day**	**1-2 h/day**	**≥2 h/day**
Folate intake							
Yes	Ref.	1.11(0.71,1.73)	1.58(0.87,2.86)	4.76(1.76,12.89) #	1.11(0.71,1.73)	1.58(0.87,2.86)	4.76(1.76,12.89) #
No	1.26(0.93,1.70)	2.72(1.73,4.28) $	2.27(1.31,3.91) #	8.22(3.05,22.19) $	2.16(1.37,3.41) $	1.80(1.04,3.11) *	6.54(2.42,17.65) $
ORs (95% CI) for folate intake within strata of ETS	1.26(0.93,1.70)	2.45(1.40,4.28) $	1.43(0.68,3.04)	1.73(0.44,6.82)			
RERI (95%CI):		**1.35(0.15,2.56)** *	0.43(-1.04,1.89)	3.20(-6.04,12.44)			
AP (95%CI):		**0.50(0.21,0.78)** $	0.19(-0.39,0.77)	0.39(-0.43,1.21)			
SI (95%CI):		4.67(0.75,29.09)	1.51(0.35,6.47)	1.80(0.36,8.88)			
Multiplicative scale (95%CI):		1.95(1.03,3.67) *	1.14(0.51,2.55)	1.37(0.34,5.59)			
HNS exposure							
No	Ref.	1.13(0.74,1.71)	1.76(1.08,2.88) *	3.71(1.55,8.87) #	1.13(0.74,1.71)	1.76(1.08,2.88) *	3.71(1.55,8.87) #
Yes	1.25(0.89,1.76)	2.55(1.63,3.99) $	1.98(1.03,3.81) *	12.11(3.53,41.47) $	2.04(1.24,3.35) #	1.58(0.79,3.15)	9.67(2.76,33.84) $
ORs (95% CI) for HNS within strata of ETS	1.25(0.89,1.76)	2.26(1.29,3.95) #	1.13(0.52,2.46)	3.26(0.74,14.44)			
RERI (95%CI):		1.17(-0.01,2.35)	-0.03(-1.57,1.51)	8.14(-7.00,23.29)			
AP (95%CI):		0.46(0.14,0.78) #	-0.01(-0.80,0.77)	0.67(0.19,1.15) #			
SI (95%CI):		4.08(0.67,24.86)	0.97(0.21,4.58)	3.75(0.68,20.76)			
Multiplicative scale (95%CI):		1.81(0.94,3.46)	0.90(0.39,2.10)	2.61(0.57,11.97)			
Paternal smoking							
No	Ref.	1.58(1.02,2.46) *	1.46(0.88,2.45)	3.31(1.27,8.61) *	1.58(1.02,2.46) *	1.46(0.88,2.45)	3.31(1.27,8.61) *
Yes	1.10(0.77,1.57)	1.58(1.05,2.37) *	2.29(1.25,4.23) #	10.07(3.43,29.56) $	1.43(0.89,2.32)	2.08(1.07,4.04) *	9.13(3.01,27.66) $
ORs (95%CI)	1.10(0.77,1.57)	1.00(0.58,1.72)	1.57(0.73,3.36)	3.04(0.74,12.55)			
RERI (95%CI):		-0.11(-1.05,0.84)	0.73(-0.83,2.29)	6.65(-4.54,17.84)			
AP (95%CI):		-0.07(-0.68,0.55)	0.32(-0.22,0.85)	0.66(0.18,1.14) #			
SI (95%CI):		0.84(0.19,3.68)	2.28(0.38,13.82)	3.75(0.65,21.75)			
Multiplicative scale (95%CI):		0.90(0.47,1.73)	1.42(0.61,3.29)	2.76(0.64,11.90)			

Note: * p<0.05; # p<0.01; $ p<0.001.

Supplementary Table S4 Interaction between maternal ETS exposure and parental factors on the risk of CHDs

Group	ETS		ORs (95%CI) for ETS within strata of another exposure
	**No**	**Yes**	
Folate intake [a]			
Yes	Ref.	1.34 (0.92, 1.95)	1.34 (0.92, 1.95)
No	1.25(0.92,1.70)	2.62 (1.80, 3.83) [$]	2.09 (1.44, 3.05) [#]
ORs (95%CI) for folate intake within strata of ETS	1.25(0.92,1.70)	1.95 (1.28, 2.70) [#]	
RERI (95%CI):		**1.03 (0.10, 1.95)** [*]	
AP (95%CI):		**0.39 (0.12, 0.66)** [#]	
SI (95%CI):		2.72 (0.90, 8.18)	
Multiplicative scale (95% CI):		1.56 (0.93, 2.61)	
HNS exposure [b]			
No	Ref.	1.47 (1.05, 2.05) [*]	1.47 (1.05, 2.05) [*]
Yes	1.28 (0.90, 1.80)	2.71 (1.86, 3.96) [$]	2.12 (1.36, 3.29) [$]
ORs (95%CI) for HNS within strata of ETS	1.28 (0.90, 1.80)	1.85 (1.21, 2.83) [#]	
RERI (95%CI):		0.96 (-0.08, 2.01)	
AP (95%CI):		0.36 (0.06, 0.65) [*]	
SI (95%CI):		2.29 (0.87, 6.01)	
Multiplicative scale (95% CI):		1.44 (0.84, 2.48)	
Paternal smoking [c]			
No	Ref.	1.57 (0.11, 2.22) [#]	1.57 (0.11, 2.22) [#]
Yes	1.07 (0.74, 1.53)	1.97 (1.40, 2.77) [$]	1.84 (1.20, 2.83) [#]
ORs (95%CI) for paternal smoking within strata of ETS	1.07 (0.74, 1.53)	1.25 (0.82, 1.90)	
RERI (95%CI):		0.33 (-0.49, 1.15)	
AP (95%CI):		0.17 (-0.22, 0.55)	
SI (95%CI):		1.51 (0.49, 4.67)	
Multiplicative scale (95% CI):		1.17 (0.68, 2.12)	

Note: * $p<0.05$; # $p<0.01$; \$ $p<0.001$.

[a] Adjusted by maternal age, maternal education level, residence, congenital anomalies family history, maternal HNS exposure, paternal smoking.

[b] Adjusted by maternal age, maternal education level, residence, congenital anomalies family history, maternal folate intake, paternal smoking.

[c] Adjusted by maternal age, maternal education level, residence, congenital anomalies family history, maternal folate intake, maternal HNS exposure.

Supplementary Table S5 Interaction between maternal ETS exposure and parental factors on the risk of CHDs with adjustment for selected covariates

Group	ETS		ORs (95%CI) for ETS within strata of another exposure
	No	Yes	
Folate intake [a]			
Yes	Ref.	1.53 (1.07, 2.56) *	1.53 (1.07, 2.56) *
No	1.77 (1.34, 2.34) $	3.78 (2.66, 5.37) $	2.14 (1.51, 3.02) $
ORs (95%CI) for folate intake within strata of ETS	1.77 (1.34, 2.34) $	2.48 (1.67, 3.67) $	
RERI (95%CI):		**1.48 (0.28, 2.68)** #	
AP (95%CI):		**0.39 (0.16, 0.62)** #	
SI (95%CI):		**2.15 (1.16, 3.96)** $	
Multiplicative scale (95% CI):		1.40 (0.87, 2.23)	
HNS exposure [b]			
No	Ref.	1.67 (1.22, 2.28) #	1.67 (1.22, 2.28) #
Yes	1.58 (1.15, 2.15) #	3.29 (2.32, 4.67) $	2.09 (1.39, 3.14) $
ORs (95%CI) for HNS within strata of ETS	1.58 (1.15, 2.15) #	1.97 (1.33, 2.93) #	
RERI (95%CI):		1.04 (-0.12, 2.21)	
AP (95%CI):		0.32 (0.04, 0.60) *	
SI (95%CI):		1.84 (0.94, 3.61)	
Multiplicative scale (95% CI):		1.25 (0.76, 2.07)	
Paternal smoking [c]			
No	Ref.	1.74 (1.27, 2.41) $	1.74 (1.27, 2.41) $
Yes	1.35 (0.97, 1.87)	2.58 (1.88, 3.55) $	1.92 (1.29, 2.84) #
ORs (95%CI) for paternal smoking within strata of ETS	1.35 (0.97, 1.87)	1.48 (1.01, 2.19) *	
RERI (95%CI):		0.49 (-0.44, 1.43)	
AP (95%CI):		0.19 (-0.14, 0.52)	
SI (95%CI):		1.45 (0.69, 3.06)	
Multiplicative scale (95% CI):		1.10 (0.66, 1.83)	

Note: * p<0.05; # p<0.01; $ p<0.001.

[a] Adjusted by maternal HNS exposure and paternal smoking.

[b] Adjusted by maternal folate intake and paternal smoking.

[c] Adjusted by maternal folate intake and HNS exposure.

Supplementary Table S6 Interaction between maternal ETS exposure and folate intake on the risk of CHDs with adjustment for maternal HNS exposure and paternal smoking

**Maternal folate intake**	**ETS**		**ORs (95%CI) for ETS within strata of another exposure**
	**No**	**Yes**	
SPD			
Yes	Ref.	1.41(0.96,2.09)	1.41(0.96,2.09)
No	1.77(1.30,2.40) $	3.44(2.35,5.04) $	1.95(1.34,2.83) $
ORs (95%CI) for folate intake within strata of ETS	1.77(1.30,2.40) $	2.43(1.58,3.74) $	
RERI (95%CI):		**1.26(0.07,2.45)** *	
AP (95%CI):		**0.37(0.10,0.63)** #	
SI (95%CI):		**2.06(1.02,4.16)** *	
Multiplicative scale (95% CI):		1.38(0.81,2.33)	
CTD			
No	Ref.	1.77(0.98,3.21)	1.77(0.98,3.21)
Yes	2.66(1.66,4.25) $	4.59(2.66,7.93) $	1.73(1.06,2.81) *
ORs (95%CI) for HNS within strata of ETS	2.66(1.66,4.25) $	2.59(1.44,4.65) #	
RERI (95%CI):		1.16(-0.93,3.24)	
AP (95%CI):		0.25(-0.13,0.63)	
SI (95%CI):		1.47(0.74,2.95)	
Multiplicative scale (95% CI):		0.97(0.46,2.06)	
LVOTO			
No	Ref.	1.31(0.76,2.26)	1.31(0.76,2.26)
Yes	1.83(1.20,2.78) #	3.49(2.14,5.70) $	1.91(1.20,3.05) #
ORs (95%CI) for paternal smoking within strata of ETS	1.83(1.20,2.78) #	2.66(1.51,4.68) $	
RERI (95%CI):		1.35(-0.15,2.85)	
AP (95%CI):		0.39(0.06,0.71) *	
SI (95%CI):		2.18(0.85,5.57)	
Multiplicative scale (95% CI):		1.45(0.72,2.93)	
RVOTO			
No	Ref.	1.53(0.90,2.60)	1.53(0.90,2.60)
Yes	1.93(1.26,2.96) #	4.45(2.72,7.26) $	2.30(1.45,3.66) $
ORs (95%CI) for paternal smoking within strata of ETS	1.93(1.26,2.96) #	2.91(1.70,4.98) $	
RERI (95%CI):		**1.99(0.17,3.81)** *	
AP (95%CI):		**0.45(0.17,0.72)** $	
SI (95%CI):		**2.36(1.06,5.26)** *	

Multiplicative scale (95% CI):		1.51(0.76,2.99)	
Other CHDs			
No	Ref.	1.54(0.77,3.09)	1.54(0.77,3.09)
Yes	1.59(0.89,2.85)	5.44(2.99,9.89) $	3.41(1.93,6.03) $
ORs (95%CI) for paternal smoking within strata of ETS	1.59(0.89,2.85)	3.53(1.84,6.80) $	
RERI (95%CI):		**3.31(0.69,5.92)** #	
AP (95%CI):		**0.61(0.35,0.86)** $	
SI (95%CI):		**3.92(1.16,13.17)** *	
Multiplicative scale (95% CI):		2.22(0.93,5.31)	

Note: * p<0.05; # p<0.01; $ p<0.001.

Supplementary Table S7 Interaction between maternal exposures to ETS and HNS on CHDs with adjustment for maternal folate intake and paternal smoking.

Maternal HNS exposure	ETS		ORs (95%CI) for ETS within strata of another exposure
	No	Yes	
SPD			
Yes	Ref.	1.59(1.14,2.23) #	1.59(1.14,2.23) #
No	1.53(1.09,2.16) *	2.79(1.90,4.09) $	1.82(1.16,2.85) #
ORs (95%CI) for HNS within strata of ETS	1.53(1.09,2.16) *	1.75(1.14,2.70) *	
RERI (95%CI):		0.67(-0.46,1.79)	
AP (95%CI):		0.24(-0.11,0.58)	
SI (95%CI):		1.59(0.72,3.51)	
Multiplicative scale (95% CI):		1.14(0.66,1.98)	
CTD			
No	Ref.	1.67(1.01,2.73) *	1.67(1.01,2.73) *
Yes	1.88(1.16,3.06) *	3.51(2.11,5.86) $	1.87(1.03,3.37) *
ORs (95%CI) for HNS within strata of ETS	1.88(1.16,3.06) *	2.11(1.18,3.76) *	
RERI (95%CI):		0.96(-0.83,2.76)	
AP (95%CI):		0.27(-0.15,0.70)	
SI (95%CI):		1.62(0.65,4.03)	
Multiplicative scale (95% CI):		1.12(0.53,2.38)	
LVOTO			
No	Ref.	1.77(1.13,2.75) *	1.77(1.13,2.75) *
Yes	1.73(1.10,2.73) *	2.44(1.47,4.05) $	1.41(0.79,2.52)
ORs (95%CI) for paternal smoking within strata of ETS	1.73(1.10,2.73) *	1.38(0.79,2.42)	
RERI (95%CI):		-0.06(-1.48,1.36)	
AP (95%CI):		-0.02(-0.61,0.56)	
SI (95%CI):		0.96(0.37,2.52)	
Multiplicative scale (95% CI):		0.80(0.39,1.64)	
RVOTO			
No	Ref.	1.98(1.26,3.11) #	1.98(1.26,3.11) #
Yes	2.20(1.40,3.44) $	4.10(2.55,6.59) $	1.87(1.09,3.21) *
ORs (95%CI) for paternal smoking within strata of ETS	2.20(1.40,3.44) $	2.07(1.23,3.49) #	
RERI (95%CI):		0.92(-0.99,2.83)	
AP (95%CI):		0.22(-0.18,0.63)	
SI (95%CI):		1.42(0.69,2.93)	

Multiplicative scale (95% CI):		0.94(0.47,1.87)	
Other CHDs			
No	Ref.	2.35(1.34,4.13) #	2.35(1.34,4.13) #
Yes	1.79(0.96,3.35)	4.85(2.74,8.59) $	2.71(1.35,5.42) #
ORs (95%CI) for paternal smoking within strata of ETS	1.79(0.96,3.35)	2.07(1.12,3.80) *	
RERI (95%CI):		1.71(-0.84,4.26)	
AP (95%CI):		0.35(-0.05,0.76)	
SI (95%CI):		1.80(0.75,4.31)	
Multiplicative scale (95% CI):		1.15(0.48,2.76)	

Note: * $p<0.05$; # $p<0.01$; $ $p<0.001$.

Supplementary Table S8 Interaction between maternal ETS exposure and paternal smoking on the risk of CHDs with adjustment for maternal folate intake and HNS exposure

Paternal smoking	ETS		ORs (95%CI) for ETS within strata of another exposure
	No	Yes	
SPD			
Yes	Ref.	1.70(1.19,2.42) [#]	1.70(1.19,2.42) [#]
No	1.41(0.99,2.02)	2.30(1.63,3.26) [$]	1.63(1.06,2.50) [*]
ORs (95%CI) for folate intake within strata of ETS	1.41(0.99,2.02)	1.35(0.89,2.07)	
RERI (95%CI):		0.19(-0.78,1.16)	
AP (95%CI):		0.08(-0.33,0.49)	
SI (95%CI):		1.17(0.51,2.66)	
Multiplicative scale (95% CI):		0.96(0.55,1.67)	
CTD			
No	Ref.	1.60(0.95,2.69)	1.60(0.95,2.69)
Yes	1.37(0.82,2.29)	2.67(1.68,4.25) [$]	1.95(1.08,3.50) [*]
ORs (95%CI) for HNS within strata of ETS	1.37(0.82,2.29)	1.67(0.93,2.99)	
RERI (95%CI):		0.70(-0.68,2.08)	
AP (95%CI):		0.26(-0.20,0.72)	
SI (95%CI):		1.72(0.52,5.70)	
Multiplicative scale (95% CI):		1.22(0.56,2.65)	
LVOTO			
No	Ref.	1.95(1.22,3.11) [#]	1.95(1.22,3.11) [#]
Yes	1.88(1.19,2.96) [#]	2.40(1.52,3.81) [$]	1.28(0.75,2.20)
ORs (95%CI) for paternal smoking within strata of ETS	1.88(1.19,2.96) [#]	1.23(0.71,2.13)	
RERI (95%CI):		-0.42(-1.86,1.01)	
AP (95%CI):		-0.18(-0.81,0.46)	
SI (95%CI):		0.77(0.32,1.84)	
Multiplicative scale (95% CI):		0.66(0.32,1.34)	
RVOTO [a]			
No	Ref.	1.51(0.94,2.45)	1.51(0.94,2.45)
Yes	1.14(0.69,1.88)	3.05(2.01,4.64) [$]	2.69(1.54,4.70) [$]
ORs (95%CI) for paternal smoking within strata of ETS	1.14(0.69,1.88)	2.02(1.18,3.44) [*]	
RERI (95%CI):		**1.40(0.09,2.72)** [*]	
AP (95%CI):		**0.46(0.13,0.79)** [#]	
SI (95%CI):		3.16(0.73,13.70)	

Multiplicative scale (95% CI):		1.78(0.85,3.69)	
Other CHDs [a]			
No	Ref.	1.94(1.06,3.53) *	1.94(1.06,3.53) *
Yes	1.20(0.61,2.35)	4.16(2.49,6.97) $	3.48(1.72,7.06) $
ORs (95%CI) for paternal smoking within strata of ETS	1.20(0.61,2.35)	2.15(1.14,4.04) *	
RERI (95%CI):		2.03(-0.01,4.07)	
AP (95%CI):		0.49(0.13,0.84) #	
SI (95%CI):		2.79(0.79,9.83)	
Multiplicative scale (95% CI):		1.80(0.71,4.51)	

Note: * p<0.05; # p<0.01; $ p<0.001.
